# Supplementary material for: How to estimate health service coverage in 58 districts of Benin with no survey data: Using hybrid estimation to fill the gaps
Source: PLOS Glob Public Health. 2022 May 25;2(5):e0000178. doi: 10.1371/journal.pgph.0000178 (PMC10022106; doi:10.1371/journal.pgph.0000178)
Supplement: S1 Text — Description: Further details outlining the Bayesian approach described in the methodology section. (DOCX) [file pgph.0000178.s001.docx]

**S1 Text.**

**Details of the Bayesian Approach and Truncation of the Posterior Predictive**

In the Methods section, we describe taking a Bayesian approach, whereby we treat the denominators $d_{j}$ for $j=20,\ldots,77$ as random variables with probability distributions centered at the predicted estimates $\hat{d}_{j}$ with variances consistent with an out of sample prediction interval ($\sigma_{\hat{d}_{j}}^{2})$. For example, we could use the normal model, with the $d_{j}$ independent:

$$d_{j}\sim N(\hat{d}_{j},\sigma_{\hat{d}_{j}}^{2})$$

If $f^{*}(d_{j})$ is the density constructed above for the denominator in commune $j$ then we truncate by the numerator as follows:

$$f\left( d_{j} \right)=\frac{f^{*}(d_{j})I(d_{j}>n_{j})}{\int_{n_{j}}^{\infty} f^{*}(\theta)d\theta}$$

This ensures the proper support for the density function by making explicit that we are not willing to consider denominators less than the number of children vaccinated in a given commune. This also ensures that our estimate/density of $p_{j}$ is less than 1. To get the posterior predictive density for $p_{j}$ we simply divide the administrative numerator $n_{j}$ by random draws from the posterior predictive density $f\left( d_{j} \right)$. Then we can use the median of these draws for point estimates of vaccination rates and the 2.5% and 97.5% quantiles for credible intervals.
