# Supplementary material for: How to estimate health service coverage in 58 districts of Benin with no survey data: Using hybrid estimation to fill the gaps
Source: PLOS Glob Public Health. 2022 May 25;2(5):e0000178. doi: 10.1371/journal.pgph.0000178 (PMC10022106; doi:10.1371/journal.pgph.0000178)
Supplement: S2 Text — Description: The sensitivity analysis performed assuming a varying numerator in the communes. An outline of the approach for the sensitivity analysis and observed changes in the prevalence estimates assuming different numerators are presented. (DOCX) [file pgph.0000178.s002.docx]

**S2 Text.**

**Bayesian Sensitivity Analysis for Predicted Denominators**

Say we have the probability density of $f\left( d_{j} \right)$. This estimate may be too high or too low, but by introducing sensitivity parameters(18) we can shift this distribution and see how our inferences might change. Consider the sensitivity parameters $\Delta_{j1}$ for the mean and $\Delta_{j2}$ for the variance of the posterior predictive distribution in commune $j$. Then we can easily alter the density of the coverage as follows:

$$d_{j}\sim N(\hat{d}_{j}+\Delta_{j1},\Delta_{j2}\sigma_{\hat{d}_{j}}^{2})$$

In addition, sensitivity to the numerator can also be incorporated by introducing a third sensitivity parameter $\boldsymbol{\Delta}_{3}$. We can vary our choices of these sensitivity parameters, perform the truncation using ${n_{j}\times\Delta}_{j3}$, and conduct inferences as above. Such a sensitivity analysis is important especially when the model is based on observed data that may not be representative of the country as a whole. Adjusting $\boldsymbol{\Delta}_{j3}$ can prove useful in considering bias in our administrative numerator.

We now conduct a sensitivity analysis on the Benin data by varying three sensitivity parameters $\boldsymbol{\Delta}_{1}, \boldsymbol{\Delta}_{2},$ and $\boldsymbol{\Delta}_{3}$. These sensitivity parameters are J dimensional, where J is the number of administrative regions for which we are extrapolating results. We therefore write $\boldsymbol{\Delta}_{kj}$ to denote the $k$th sensitivity parameter in commune $j$, although in subsequent sensitivity analysis, we will consider a constant value for $\boldsymbol{\Delta}_{k}$ across all communes.

We now describe and present results from a sensitivity analysis of the administrative numerator in the communes without probability survey data. This corresponds to tuning the sensitivity parameter $\boldsymbol{\Delta}_{3}$. This approach would be analogous for investigating the sensitivity of the estimated denominators and the variance of their estimation (i.e. $\boldsymbol{\Delta}_{1}$ and $\boldsymbol{\Delta}_{2}$). Note that when $\boldsymbol{\Delta}_{1}\neq1$ we explicitly capture departures from the missing at random (MAR) assumption.

Recall that when $\boldsymbol{\Delta}_{3}\neq1$ we are assuming that our administrative numerator is biased and must be adjusted. The sensitivity parameter $\boldsymbol{\Delta}_{3}$ has a multiplicative effect on our observed administrative numerator $n_{j}$ to form a new estimate of the numerator $\zeta_{3}$:

$$\zeta_{3}{=n_{j}\times\boldsymbol{\Delta}}_{j3}$$

Then by using this new adjusted numerator we can proceed with the posterior truncation and transformation in the same way as described above.

We considered scenarios where the observed administrative numerator $n_{j}$ was 5% and 10% biased in both directions (i.e. -10%,-5%,5% ,10%). This corresponds to choosing values of the sensitivity parameter in the set $\boldsymbol{\Delta}_{3}\in[0.9,0.95,1.05,1.1]$. The resulting estimates and confidence intervals are shown in Figure S2.1:


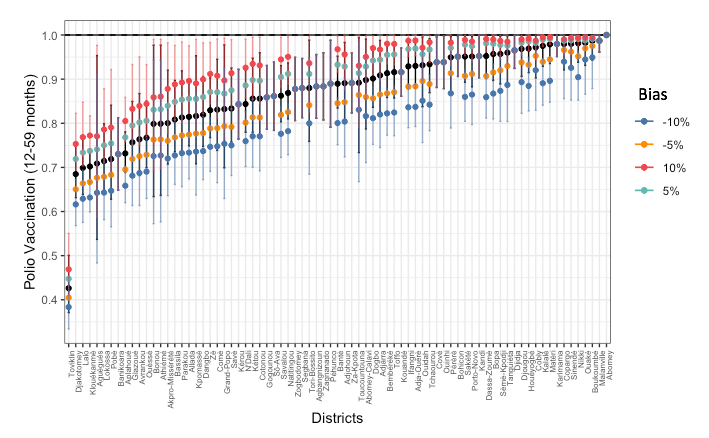


**Figure S2.1.** Sensitivity Analysis of Administrative Numerator for the Polio Vaccination Coverage among 12-59 month old children

We see the original estimates in black and the sensitivity estimates for the varying choices of our sensitivity parameter labelled by the figure legend. We see the bias to be fairly equal in both directions on the left side of the plot, but as we approach the upper margin the upward bias becomes negligible. This is a result of our truncation of the posterior. We find inferences to be fairly robust despite 10% bias in the administrative numerator.

The above approach is actually analogous to the frequentist approach for a sensitivity analysis. We could alternatively take advantage of the fact that we are in the Bayesian paradigm to build priors on our sensitivity parameters instead of choosing arbitrary values as above. In fact, the frequentist sensitivity analysis corresponds to summarizing conditional posteriors, where conditioning is done on fixed values of $\boldsymbol{\Delta}$ as shown above. By postulating a prior for $\boldsymbol{\Delta}$ we could account for a smooth gradient in the bias rather than simply consider discrete values of $\boldsymbol{\Delta}$.

Consider using the prior $\boldsymbol{\Delta}_{j3}\sim N(1,0.1)$ to quantify our belief about the bias in our administrative numerator. Because we have adopted a Bayesian framework we then simply incorporate the added variability/bias of this prior into our posterior predictive distribution. The results are shown in Figure S2.2:


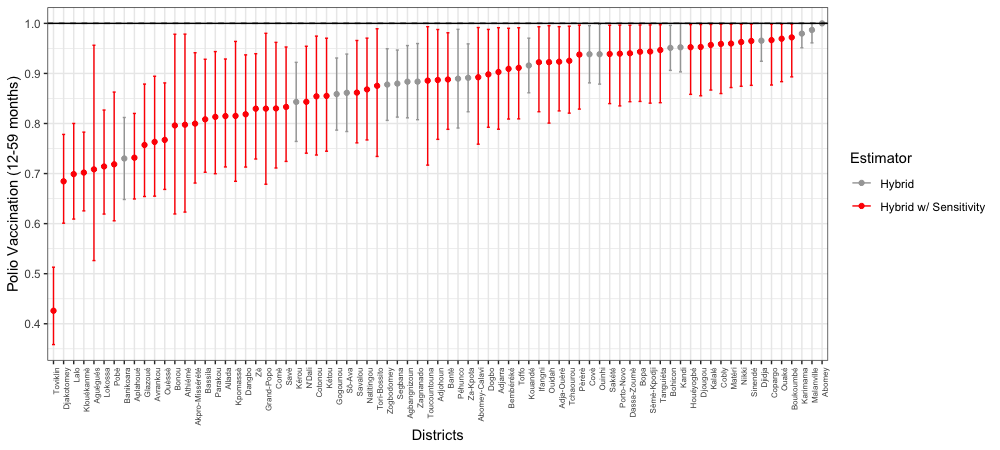


**Figure S2.2.** Bayesian Sensitivity Analysis of Administrative Numerator for the Polio Vaccination Coverage among 12-59 month old children with $\boldsymbol{\Delta}_{j3}\sim N(1,0.1)$ prior

This particular choice of prior doesn’t change the point estimates, because it is centred at “no bias”. However, the credible intervals of our posterior predictive distributions in our sensitivity analysis are wider than in the original Bayesian analysis, reflecting that we are taking into account the added variability due to uncertainty in the administrative numerator.

Analysts should note that the truncation may have effects downstream on the effect estimates and variance of the final parameters once $\Delta_{j1}$ and $\Delta_{j2}$ are specified. This is an important to note, and consequently the analyst should make sure to consider a wide enough range of sensitivity parameters $\Delta_{j1}$ and $\Delta_{j2}$ relative to the sensitivity numerator $\zeta_{3}$ used for truncation.
