# Supplementary material for: How to estimate health service coverage in 58 districts of Benin with no survey data: Using hybrid estimation to fill the gaps
Source: PLOS Glob Public Health. 2022 May 25;2(5):e0000178. doi: 10.1371/journal.pgph.0000178 (PMC10022106; doi:10.1371/journal.pgph.0000178)
Supplement: S3 Text — Description: Results for the three other health indicators and populations using the same methodology presented in the main text are included. (DOCX) [file pgph.0000178.s003.docx]

**S3 Text.
 Results for Other Health Indicators and Populations in Benin**

Both administrative data and LQAS surveys are available for Vitamin A supplementation as well as for Polio. In addition, coverage estimates are generally stratified by children aged 6-11 months as well as those 12-59 months. We showcase the additional results here. The first set of Figures (S3.1-3) present the results of the frequentist analysis.


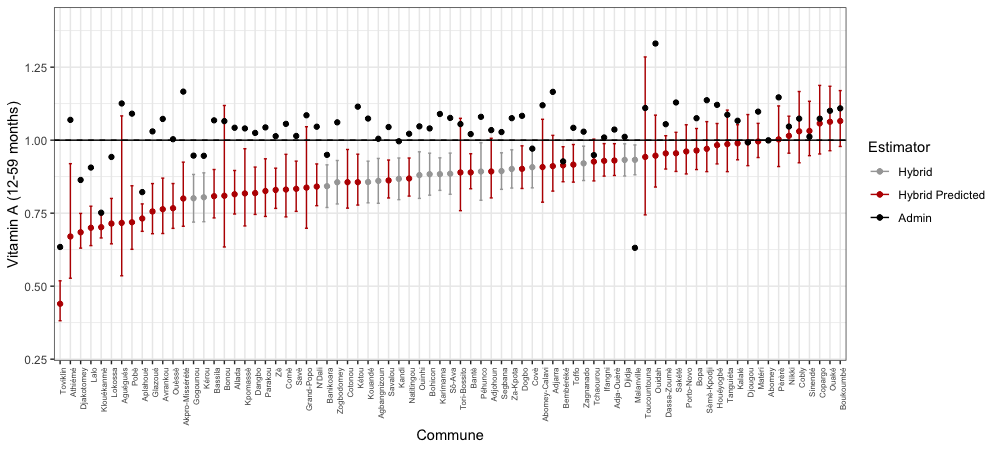

**Figure S3.1.** Vitamin A supplementation coverage among 12-59 month old children


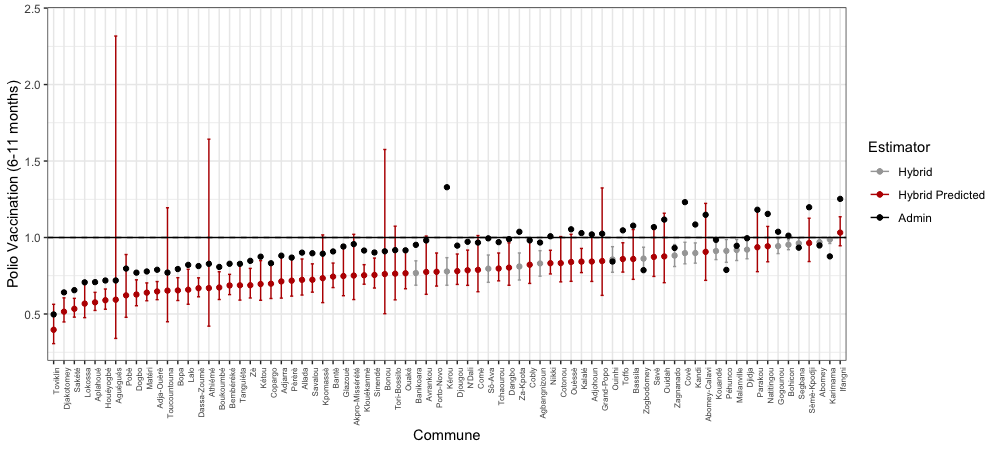

**Figure S3.2.** Polio vaccination coverage among 6-11 month old children


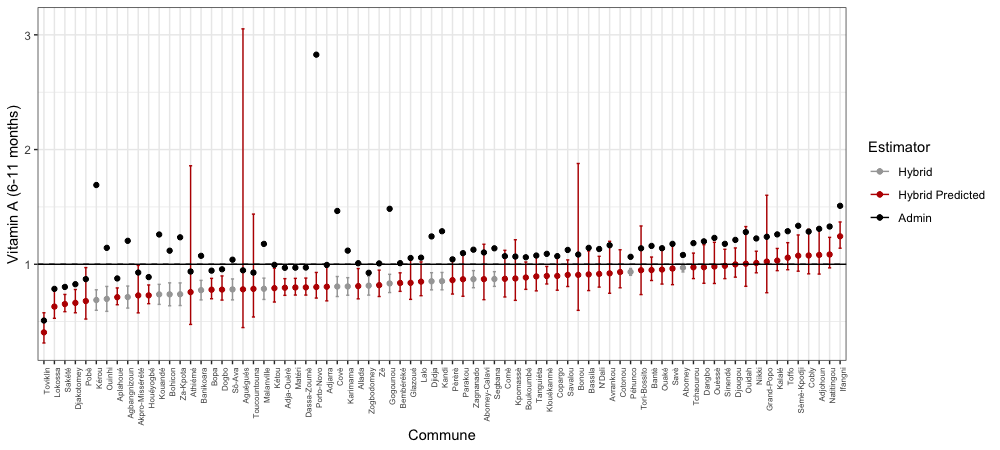


**Figure S3.3.** Vitamin A supplementation coverage among 6-11 month old children

Figures S3.4-6 present the resulting Bayesian estimates for the same above indicators. We note the Bayesian adjustment forces estimates and intervals to fall in the appropriate ranges (i.e. below 1).

Bayesian Results


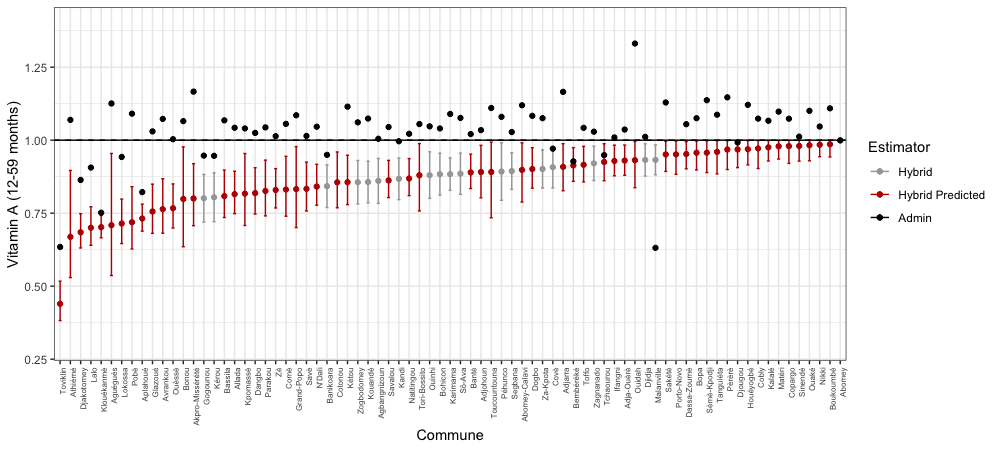


**Figure S3.4.** Vitamin A supplementation coverage among 12-59 month old children


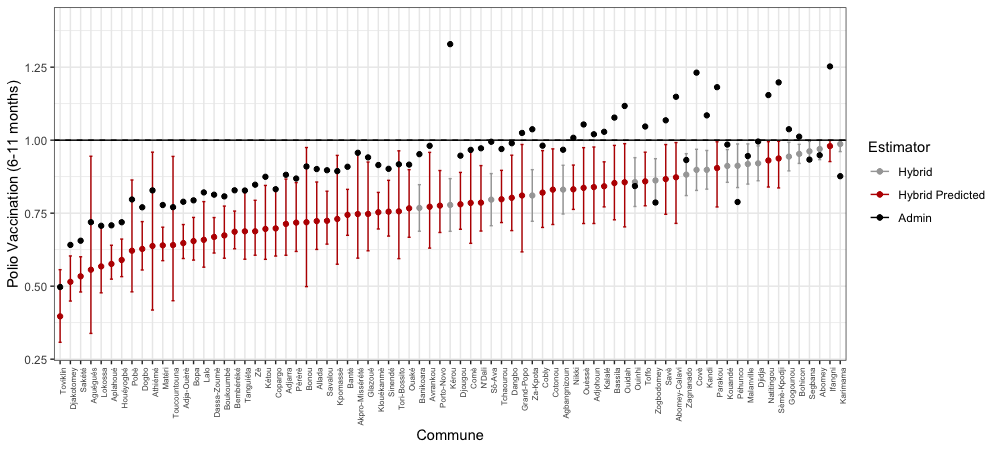


**Figure S3.5.** Polio vaccination coverage among 6-11 month old children


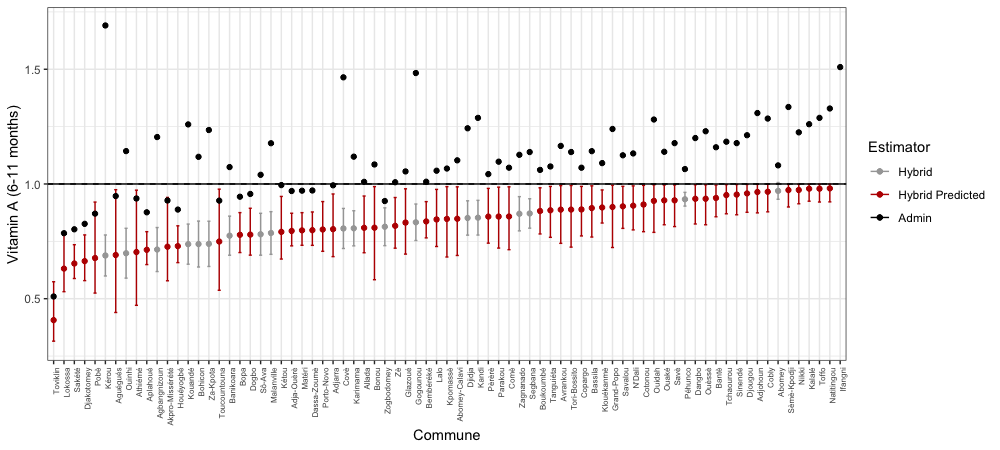


**Figure S3.6.** Vitamin A supplementation coverage among 6-11 month old children
