# Supplementary material for: How to estimate health service coverage in 58 districts of Benin with no survey data: Using hybrid estimation to fill the gaps
Source: PLOS Glob Public Health. 2022 May 25;2(5):e0000178. doi: 10.1371/journal.pgph.0000178 (PMC10022106; doi:10.1371/journal.pgph.0000178)
Supplement: S4 Text — Description: Results using the negative binomial distribution. (DOCX) [file pgph.0000178.s004.docx]

**S4 Text.
 Results for a Negative Binomial Bayesian Model**

In the main manuscript, a Normal model was postulated for the out of sample predictions of the denominators in communes without survey data. One reviewer pointed out that this may not be flexible enough, and an alternative parameterization such as a negative binomial model may be more appropriate. Herein, we present results using the negative binomial distribution.

Firstly, we consider the negative binomial distribution with parameterization:

$$P\left( d_{j}=x \right)=\frac{\Gamma\left( x+s \right)}{\Gamma\left( s \right)}p^{s}\left( 1-p \right)^{x}$$

We do this to be explicit because there are numerous parameterizations of the negative binomial distribution. Next, we postulate the following model:

$$d_{j}\sim NB\left( s=\frac{\hat{d}_{j}^{2}}{\left( \sigma_{d_{j}}^{2}-\hat{d}_{j} \right)},p=\frac{\hat{d}_{j}}{\sigma_{d_{j}}^{2}} \right)$$

Where $\hat{d}_{j}$ are the mean predictions from the postulated regression model and $\sigma_{d_{j}}^{2}$ the out of sample variances for commune $j$. Now that each negative binomial distribution for the denominators is defined, we truncate this distribution by the known numerator as before:

$$f\left( d_{j} \right)=\frac{f^{*}(d_{j})I(d_{j}>n_{j})}{\int_{n_{j}}^{\infty} f^{*}(\theta)d\theta}$$

To obtain the final posterior distribution for $d_{j}$. The results are presented in Figure S4.1.


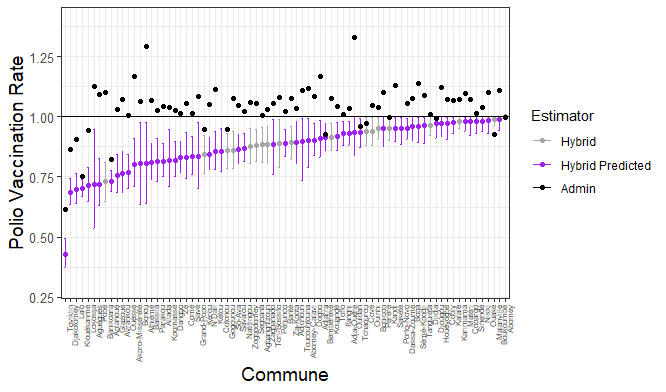


**Figure S4.1.** Polio Vaccination coverage among 12-59 month old children using a Negative Binomial Model

The results are nearly identical to those of the normal model. Why is this? It is a well known fact that a negative binomial distribution is approximately normal when s is sufficiently large. This is because a negative binomial is in a sense, a sum of independent geometrically distributed random variables. Therfore, by Central Limit arguments, the negative binomial distribution with large s converges in distribution to a normal distribution. In our data application, s is quite large because the denominators are large numbers. We noticed that for communes with larger denominators, and subsequently large s, the normal results and negative binomial results are nearly identical. For smaller communes, the results are slightly different. However, overall, the s parameters of the negative binomial distributions considered is still large enough to warrant a normal approximation.
