# Supplementary material for: How to estimate health service coverage in 58 districts of Benin with no survey data: Using hybrid estimation to fill the gaps
Source: PLOS Glob Public Health. 2022 May 25;2(5):e0000178. doi: 10.1371/journal.pgph.0000178 (PMC10022106; doi:10.1371/journal.pgph.0000178)
Supplement: S5 Text — Description: This sensitivity analysis reveals that reducing the predicted denominators to one covariate decreases accuracy of the “hybrid predicted” estimates (i.e. wider confidence intervals). (DOCX) [file pgph.0000178.s005.docx]

**S5 Text.
 Results for Estimating Denominators using only Population Data**

As a sensitivity analysis, we performed the approach using only one covariate - the populations from the 2013 Census in Benin – in the regression model to estimate denominators.


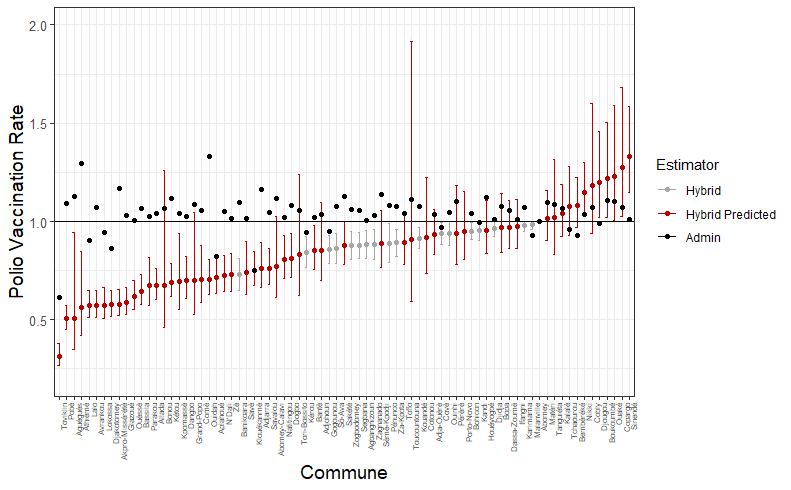


**Figure S5.1.** Polio vaccination coverage among 12-59 month old children (Frequentist)

This sensitivity analysis reveals that reducing the predicted denominators to one covariate decreases accuracy of the “hybrid predicted” estimates (i.e. wider confidence intervals). Therefore, the estimates additionally bounce around more and we see a much wider range of coverage estimates as compared to the original analysis (Figure 3 in main manuscript). The same finding holds for the Bayesian analysis.


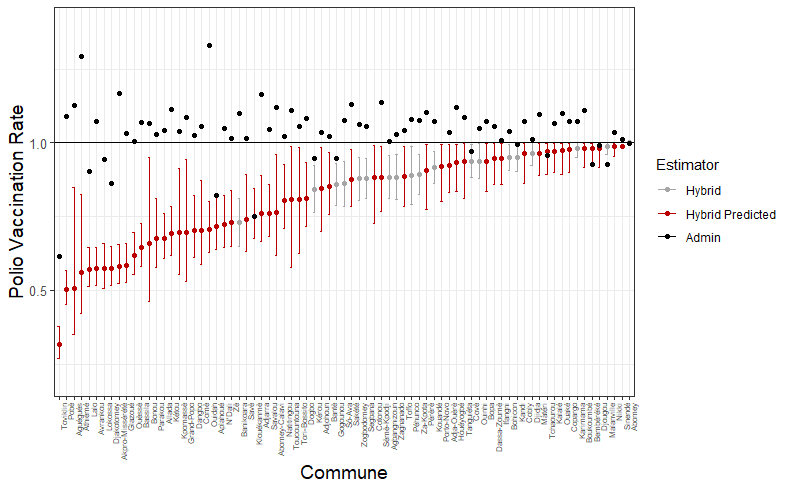


**Figure S5.2.** Polio vaccination coverage among 12-59 month old children (Bayesian)

These findings make the case for including even more covariates to increase the accuracy of the predicted denominators as well as the efficiency of the coverage estimates.
