# Supplementary material for: How to estimate health service coverage in 58 districts of Benin with no survey data: Using hybrid estimation to fill the gaps
Source: PLOS Glob Public Health. 2022 May 25;2(5):e0000178. doi: 10.1371/journal.pgph.0000178 (PMC10022106; doi:10.1371/journal.pgph.0000178)
Supplement: Attachment — Submitted filename: Response to Reviewers_FINAL.docx [file pgph.0000178.s007.docx]

**To the Editors:**

We are grateful to the editor and the reviewers for their comments and insights. Please find our responses below.

**Response to Reviewers:**

**How to estimate health service coverage in 58 districts of Benin with no survey data: using hybrid estimation to fill the gaps**

Journal Requirements:

1. Please include a complete copy of PLOS’ questionnaire on inclusivity in global research in your revised manuscript. Our policy for research in this area aims to improve transparency in the reporting of research performed outside of researchers’ own country or community. The policy applies to researchers who have travelled to a different country to conduct research, research with Indigenous populations or their lands, and research on cultural artefacts. The questionnaire can also be requested at the journal’s discretion for any other submissions, even if these conditions are not met.  Please find more information on the policy and a link to download a blank copy of the questionnaire here: <https://journals.plos.org/globalpublichealth/s/best-practices-in-research-reporting>. Please upload a completed version of your questionnaire as Supporting Information when you resubmit your manuscript.

We include our responses to the questionnaire as “Supplementary Information 6: Inclusivity Questionnaire.”

2. In the online submission form, you indicated that "The probability survey datasets generated and/or analysed during the current study are available from the corresponding author on reasonable request. The administrative data is owned by UNICEF Benin from whom a request should be made."
All PLOS journals now require all data underlying the findings described in their manuscript to be freely available to other researchers, either 1. In a public repository, 2. Within the manuscript itself, or 3. Uploaded as supplementary information.
This policy applies to all data except where public deposition would breach compliance with the protocol approved by your research ethics board. If your data cannot be made publicly available for ethical or legal reasons (e.g., public availability would compromise patient privacy), please explain your reasons by return email and your exemption request will be escalated to the editor for approval. Your exemption request will be handled independently and will not hold up the peer review process, but will need to be resolved should your manuscript be accepted for publication. One of the Editorial team will then be in touch if there are any issues.

**Authors’ Response:** Upon publication we can make the anonymized Benin LQAS survey data freely available on the website of the Liverpool School of Tropical Medicine under Creative Commons Attribution Non-Commercial No Derivatives License 4.0 (CC BY-NC-ND). However, the administrative data is Health Management Information System owned by the Ministry of Health of Benin and owned by the government on behalf of the Beninois. We are legally not able to make this data available, treating it as though we were its owners.

3. Please provide us with a direct link to the base layer of the map used in figure 1 and ensure this location is also included in the figure legend.

Please note that, because all PLOS articles are published under a CC BY license ([creativecommons.org/licenses/by/4.0/](http://creativecommons.org/licenses/by/4.0)), we cannot publish proprietary maps such as Google Maps, Mapquest or other copyrighted maps. If your map was obtained from a copyrighted source please amend the figure so that the base map used is from an openly available source.

Please note that only the following CC BY licences are compatible with PLOS licence: CC BY 4.0, CC BY 2.0  and CC BY 3.0, meanwhile such licences as CC BY-ND 3.0 and others are not compatible due to additional restrictions. If you are unsure whether you can use a map or not, please do reach out and we will be able to help you.

The following websites are good examples of where you can source open access or public domain maps:

* U.S. Geological Survey (USGS) - All maps are in the public domain. ([http://www.usgs.gov](http://www.usgs.gov/))

* PlaniGlobe - All maps are published under a Creative Commons license so please cite “PlaniGlobe, [http://www.planiglobe.com](http://www.planiglobe.com/), CC BY 2.0” in the image credit after the caption. (<http://www.planiglobe.com/?lang=enl>)

* Natural Earth - All maps are public domain. ([http://www.naturalearthdata.com/about/terms-of-use/](http://www.naturalearthdata.com/about/terms-of-use))

**Authors’ Response:** This license statement was contained in the shapefile download: “These data were extracted from the GADM database (www.gadm.org), version 2.5, July 2015. They can be used for non-commercial purposes only. It is not allowed to redistribute these data, or use them for commercial purposes, without prior consent.” Should this not suffice, we are happy to remove the figure as it is not central to the results of our manuscript.

Additional Editor Comments (if provided):

We thank you for submitting your manuscript to PLOS Global Public Health. I have reviewed the feedback from both reviewers, and have also reviewed the article myself.

Both reviewers agreed that the paper addresses a very important issue in global health measurement: the ability to predict denominators to estimate population coverage levels from administrative data. I strongly agree with the reviewers on this point.

However, both reviewers have also raised some important statistical concerns about the methods used and therefore I would encourage you to consider their feedback in your revision of your manuscript.

**Authors’ Response:** We thank the editor for their comments and have done our best to address the statistical concerns of the two reviewers throughout this document. We carefully considered their commentary and found this quite useful as it allowed us to explore our approach more deeply and also perform a number of additional analyses to support the robustness of our new method.

I would also add another concern that was not directly raised by the reviewers, which is that the authors frame the introduction about the need to make better use of routine health information system data (also referred to as "recurrent" a term I have not seen widely used and that I find confusing), yet the data they use comes from campaign data, which while administrative is not necessarily "routine" by many definitions. Could the authors comments on how this many affect the generalisability of the findings?

**Authors’ Response:** We have replace the word “recurrent” with “routine” in the entire manuscript. Tally sheet data falls within the accepted definition of routine data. MEASURE Evaluation defined it (as have others) as “Routine health information systems (RHIS) comprise data collected at regular intervals at public, private, and community-level health facilities and institutions and health programs. The data give a picture of health status, health services, and health resources. Most of the data are gathered by healthcare providers as they go about their work, by supervisors, and through routine health facility surveys. The sources of those data are generally individual health records, records of services delivered, and records of health resources.” Child Health Day tally sheet data is collected at 6 monthly intervals at the community level to manage service delivery.

Also, stylistically, could the authors avoid calling their approach "innovative" and "novel" as these are highly subjective terms? I think "new" is sufficient.

**Authors’ Response:** Per the editor’s request, any mention of novel or innovative has been either removed or replace with “proposed” or “new”.

**W**e hope our revisions and clarifications help strengthen the message of our paper and look forward to any further correspondence.

Reviewer #1: The authors developed a hybrid method to estimate the denominators in all catchment areas by combining administrative data with random probability survey data available in only certain parts of the population. A Bayesian approach with Normal model was used. This denominator-generating model was also truncated to be above the observed number of vaccinations given (the numerators) so that the resultant vaccine coverage would never exceed 1. The authors also conducted a sensitivity analysis and an application to estimate the commune-level polio vaccination coverages among children aged between 12-59 months in Benin, where only 19 out of the 58 communes had survey data.

The issue that the manuscript aims to address is a very common and important question in many low- and middle-income countries; hence it is very meaningful. The manuscript was easy to read and follow, and it is accompanied with clear descriptions and graphs in most parts. However, there are some concerns for the authors to consider.

Major concerns:
1. The authors proposed a Bayesian approach where the denominators were assumed to follow a Normal model. However, Normal assumes a symmetric distribution of the data around the mean. Did the authors attempt a more flexible distribution such as a negative binomial? It seems more plausible for modelling counts data, too.

**Authors’ Response:** We appreciate the proposal to implement a more flexible distribution such as the negative binomial distribution in the Bayesian approach. We agree that such a distribution is certainly more appropriate for count data and our denominators are certainly counts. You will find the results of this analysis using the negative binomial distribution in “S4 supporting information”. We have added to the conclusion of the results section, “Lastly, a negative binomial model was further considered for the denominators in place of the normal model. The results using this alternative distribution are presented in S4 Supporting Information.”

At first surprising, the results are nearly identical to those of the normal model. Further investigation led us to a fundamental principle of probability: that as $s$, the parameter representing the number of “failures” before the experiment is stopped in the negative binomial model, approaches infinity, then the negative binomial distribution converges in distribution to a normal distribution. This is due to a Central Limit type argument. Because our denominators are quite large (relative to considering a set number of experiments) using the negative binomial distribution yields results similar to the normal model.

2. Directly truncating the denominator estimates would make the left-over Normal model have a higher mean and smaller variances compared to what the authors specified in the original inputs. Without a proper adjustment, this will cause the ratio estimate in the end to bias downwards because you were sampling your denominators from a distribution with its mean larger than what you expected. The authors should investigate the potential bias of their ratio estimators and discuss the influence of such.

**Authors’ Response:** We fully agree with the reviewer, the truncation of the normal distribution will certainly shrink the variance of the estimator. We actually see this as an advantage of our approach: incorporating knowledge of the numerator makes us more confident in plausible values of the denominator; as a consequence, this shrinks our uncertainty. Additionally, one can think of the truncation as a prior on the denominator that gives zero probability to parameter values below the numerator, which we feel is a reasonable path to take given the numerator gives us a lower bound on the number of children in a given commune in Benin. The beauty of this Bayesian approach is that we can see quantitatively how much our prior knowledge decreases this uncertainty.

3. Similar to point 2, a truncated Normal also inflates Δ_j1 and deflates Δ_j2 in the sensitivity analysis. The former case may be less of a concern but the latter one should be addressed appropriately.

**Authors’ Response:** The sensitivity parameters Δ_j1 and Δ_j2 are fixed by the analyst; therefore, they cannot be inflated or deflated by the model per se. However, we agree that the truncation may have effects downstream on the effect estimates and variance of the final parameters once Δ_j1 and Δ_j2 are chosen. This is an important to note, and consequently the analyst should make sure to consider a wide enough range of sensitivity parameters Δ_j1 and Δ_j2 relative to the sensitivity numerator $\zeta_{3}$ used for truncation.

To highlights this, we have added language to the description of the sensitivity analysis at the end of “S2 supporting info” in order to highlight this important consideration.

4. In the estimation of denominator means through a simple linear regression, the vaccination tallies data at time t-1 were used as one commune factor. But the time t vaccination tallies were then used as the numerator, which leads to a concern of autocorrelation in the vaccination data.

**Authors’ Response:** We thank the reviewer for bringing up the issue of auto-correlation between the past and future vaccination tallies (numerators), which we had not considered. We were thankful for the opportunity to explore the issue of autocorrelation more and see how it may affect our context. Firstly, we checked the residual plots of the regression for evidence of autocorrelation:

We do not see any strong evidence of autocorrelation in our analysis. Additionally, the Durbin-Watson test statistic fails to reject for evidence of autocorrelation (T= -0.083, p = 0.486).

We believe this is because our problem is not a classic autocorrelation of observations. This takes place when you have a postulated model:

$$y_{t}=\beta_{0}+\beta_{1}y_{\left\{ t-1 \right\}}+\epsilon$$

And your data uses the previous observations outcome as the predictor, such as in time series data. An example might be the following:

| Obs | Yt | Yt-1 |
| --- | --- | --- |
| 1 | 5 | 4 |
| 2 | 4 | 3 |
| 3 | 3 | 2 |
| ... | ... | ... |
| 99 | 6 | 3 |
| 100 | 3 | 4 |

Whereas in our data, the data from the lagged time used as a covariate is not an outcome in a corresponding row. The covariate for each observation is well correlated with the outcome, which in our case is an advantage. Additionally, the number of children vaccinated in the past is not equivalent to the outcome in the past; rather, the outcome of our regression is a ratio of the number of children vaccinated and the coverage probability obtained from the survey. Therefore, we do not see autocorrelation as an issue in this analysis, although it was nevertheless a fruitful exercise for our own edification to investigate the issue and make sure it was not an issue in our modeling efforts.

5. Another maybe more intuitive specification is to directly model the conditional probability of polio vaccine coverages, that is, p_j |n_j,X_j ∼ Beta(α_j, β_j), where α_j and β_j can be estimated using the 19 communes similar to what the authors had done, and X_j’s are the two commune factors. In this case, n_j's can be modelled marginally using a Poisson or a simple linear regression, while taking into account the autocorrelation altogether.

Two potential advantages of doing it this way are: 1) it automatically requires the outcome ratio to be between 0 and 1 so it avoids the “truncation bias”; and 2) Beta allows more flexibility in the shape than Normal does. I would like to see how this approach performs compared to the authors' original one.

**Authors’ Response:** We thank the reviewer for proposing to model the polio vaccination rates directly using the beta distribution. Modeling the prevalences is a logical starting point. We had considered a direct model initially as well; however, we had not considered the beta model, so we were also interested to see how this performs.

Using the beta model, we regressed the probability of polio vaccination against the same covariates of the original model. The results from the beta regression are shown in the figure below.

The differences between the beta approach and our proposed denominator strategy actually highlight the advantage of the ratio approach, which is attributed to a senior author of our paper and draws its inspiration from the work of Laplace centuries ago. Using ratios as a form of extrapolation can be very powerful when the ratio estimated is interpretable and provides advantages over direct modeling of specific outcomes. For instance, in order for a direct model such as the proposed beta model to work, we would need a rich set of covariates that are associated with polio vaccination rates (e.g. social determinants of health, healthcare infrastructure, trust in vaccinations, etc.). Our approach is an easier problem because the ratio represents a denominator that is reflective of child population in each commune. Therefore, a simpler set of covariates indicative of the size of each commune (i.e. census data etc.) are sufficient to get a good model fit and dependable extrapolation. We thank the reviewer for allowing us to think through this alternative as this exercise helped to affirm our confidence in our proposed method.

Minor concerns & suggestions:
1. There appears to be only 18 points on Figure 2, which commune was missing?

**Authors’ Response:** We appreciate the catch. The commune of Abomey was accidentally omitted from the plot. It has been included in the updated figure 2 in the submission.

2. I did not fully understand how the authors came up with the variances in the normal model (page 9). Please provide more details.

**Authors’ Response:** We appreciate the request for further details on the variances used in the normal model. The reviewer will find a more detailed methods section to address this clarification as well as describe our modeling choices more clearly. These variances use the variance of the residual error term from the regression model to calculate the out of sample prediction variance which is denoted $\sigma_{\hat{d}_{j}}^{2}$. A Bayesian regression with uniform prior has posterior predictive $y_{new}\sim N\left( X_{new}\beta,\sigma_{\hat{d}_{j}}^{2} \right)$ and so the results from the regression are consistent with the normal model. This justifies sampling from the normal distribution as a consequence. We state, “The proposed Bayesian approach begins by postulating that the denominators $d_{j}$ for $j=20,\ldots,77$, are random variables with probability distributions centered at the previously predicted estimates $\hat{d}_{j}$ with variances consistent with an out of sample prediction interval ($\sigma_{\hat{d}_{j}}^{2})$.” We hope this helps clarify this point in the text.

3. I assume the first paragraph in the Results section deals with the method using direct regression estimates. The authors should be clearer about what they are writing here.

Thank you for this suggestion. We have edited the first 2 sentences of the Results: “The estimated polio vaccination rates and corresponding 95% confidence interval for children ages 12-59 months in each commune using the direct regression estimates are shown in (Figure 3). These include both the baseline hybrid estimators from the 19 communes (grey dots) and the extrapolated hybrid estimates (red dots).”

4. For Figures 3 and 4, are those "hybrid" grey dots the 19 communes used as the baseline? Also, it would be helpful to see the prediction intervals for those 19 communes as a proof of concept to make sure at least the model is interpolating properly.

The reviewer is correct, the 19 grey dots are the estimates used in the baseline model. We have performed the requested comparison to demonstrate that the model is interpolating properly. The black dots are the denominators fed into the regression model and the grey points & intervals are the model predictions. Here is the requested figure:

5. Although the authors clarified their use of a simple linear regression in the Discussion section, it was originally unclear what method was used when I was reading the Methods on page 9 for the first time, as it only says they “built a regression model” with no clarification in terms of the regression model forms.

**Authors’ Response:** We appreciate the request for more detailed explanation of the methodology. The updated methods section is much more explicit about the modeling to remove ambiguity. We explicitly spell out the linear model and discuss the basic assumptions to make clear it is a standard linear regression.

6. What if only the 2013 census data with adjustments of population growth rates was used as the commune factor? It may be interesting to include this as part of the sensitivity analysis to see how sensitive the estimators are to the “quality” of data that the denominator estimates are based upon. It is also possible that the population did not change as much between 2013 and 2015 so you would see almost identical results.

The proposed sensitivity analysis is welcomed. We have performed the requested sensitivity analysis and included the results in a new additional file: “S5 Supporting Info.” The analysis shows, consistent with intuition, that decreasing the covariate information increases uncertainty in the estimates. We believe this makes the case for the inclusion of a more robust set of covariates, which is suggested in the discussion of the paper. We have added to the discussion a sentence to highlight this, “A sensitivity analysis conducted using only the census data as a sole covariate resulted in less accurate estimates (S5 Supporting Information), making the case for incorporating even more covariate information.”

7. The figure labels were referenced incorrectly in Additional File 2.

**Authors’ Response:** The figures in Additional File 2 are now referenced correctly as Figure S1 and Figure S2. We have uploaded a revised Additional File 2.

Reviewer #2: This is an interesting paper, that address an important problem in global health metrics: how to obtain coverage indicators from health facility/health service data. The authors, in particular, grapple with a vexing issue: the frequent occurrence of coverage rates > 100% due to inaccurate denominators. They propose a solution that relies on combining multiple data sources, along with Bayesian methods. I thought the paper and methods were clear, but several revisions/clarifications would improve the manuscript.

1) how frequent are LQAS surveys? this is unclear from the manuscript, but the utility of the methods depends directly on the availability of such sample surveys. Are they conducted every year? less frequently? more frequently? we are only told that they are conducted widely, and the review cited covers the period 1984-2004. Maybe a map of LMICs indicating recent LQAS data availability could be inserted (e.g., time since the last LQAS survey in each country).

There is no set interval for carrying out LQAS or other types of Knowledge, Attitude and Practice Surveys. They are used by Ministries of Health, UN- and other donor agencies, and non-governmental organizations for Programme Monitoring and Evaluation. KAP surveys (whether they are LQAS or cluster surveys) are required for most grant funding to assess whether targets are being reached. There is no current Global Review of LQAS or other KAP survey methods, although there have been more recent studies assessing their relative costs [1]. In our earlier paper which we reference in the current ms, we make the point that our Statistical Annealing Technique (AT) can use any type of probability survey or combination of sources of probability surveys [2]; LQAS survey are not a requirement but they are very convenient to use for AT because of their local level measures.

2) the ratio estimator depends critically on the accuracy of the parameter n, i.e., the number of children receiving a service or intervention. This is treated at some length in appendix 2, via sensitivity analyses, but I think at least one of the figures of that appendix should be moved to the main text, and discussed in the abstract. Otherwise, I think the reader might not accurately grasp the uncertainties that still underpin this method.

**Authors’ Response:** We agree with the reviewer that it is crucial to highlight how much our method hinges on the accuracy of the parameter n (the number of children receiving the intervention) and appreciate the suggestion to include one of the figures from the sensitivity analysis in the main body of the text. You will now find the results of the sensitivity analysis considering a numerator that is biased by -10%, -5%, 0%, 5%, and 10% in the results section of the manuscript.

3) there are also studies in LMICs that have looked at the completeness of administrative via record linkages, for example, or re-tallying of facility registers. it might be worth citing these studies.

**Authors’ Response:** In the Discussion on page 16, Lines 394-397 we have add the following text and citations to address these points you have raised: “However, bias due to the incompleteness or inaccurate data entry into records can be reduced by Data Quality Audits, Routine Data Quality Assessments, the use of electronic data entry; these approaches have been shown to improve data accuracy in lower and middle income countries [3-6] .”

4) some of the features of the model (e.g., truncation by reality) could also be applied to outdated census data (and associated projections) that underpin current estimates of coverage. In fact, it would be quite interesting to see how close to the hybrid estimates one might get with only census data as input for d + an appropriate Bayesian framework. I'm asking because such projections from census data are available for every year-area, with uncertainty increasing with time since last census and smaller areas.

**Authors’ Response:** Reviewer #1 also asked for the accuracy based on only the census data provided. We have included this in the “S6 Supporting Info”. The analysis reveals that limiting the regression to a sole covariate increases uncertainty in estimates, making the case for inclusion of even more covariates in practice. We have added to the discussion a sentence to highlight this, “A sensitivity analysis conducted using only the census data as a sole covariate resulted in less accurate estimates (S5 Supporting Information), making the case for incorporating even more covariate information.” Also, while in theory, census data is available every year for any given country, this is not necessarily broken down by the commune level in Benin. The most recent census data at the commune level for Benin we were able to find was that for 2013.

5) What is the inter-intervention of the denominator estimates? by this, i mean, that we obtained an estimate based on the coverage of vaccination, but do we get something similar based on, say, a nutrition intervention? this goes back to issues of completeness of the admin data, but it would be very interesting to learn if such an assessment is possible in Benin.

**Authors’ Response:** We welcome the suggestion to investigate consistency between the inter-intervention estimates of denominators. We have the ability to assess this, as we have data available on two indicators in Benin: 1) Vitamin A supplementation and 1) Polio Vaccination. We plotted the correlation between the two for children ages 6-11 months and those 12-59 months:

These plots are encouraging. They show that both interventions draw similar conclusions in denominator estimates. In addition, we have included more details on estimating denominators when more than one intervention is available and included this inverse variance weighted estimate in the updated methodology section of the paper.

6) i suspect the sample of the Benin survey relied on census data for parts of the selection process (e.g., maps?). This is unclear to me from the text, or from the references cited. it would help to have that detail. If census data is used at any stage, how is the uncertainty it carries accounted for in the model?

**Authors’ Response:** The Benin household survey used probability proportional to size to sample villages with commune as interview locations. The sampling frame for the PPS was provided by UNICEF using data provided by the Ministry of Health’s Direction de la Planification et de la Prospective. No census maps were used. Rather maps were made by data collectors of each sampled village for segmentation sampling which led the interviewer to a randomly selected household.

References

1.	Anoke SC, Mwai P, Jeffery C, Valadez JJ, Pagano M. Comparing two survey methods of measuring health-related indicators: Lot Quality Assurance Sampling and Demographic Health Surveys. Tropical Medicine & International Health. 2015;20(12):1756-70. doi: 10.1111/tmi.12605.

2.	Jeffery C, Pagano M, Hemingway J, Valadez JJ. Hybrid prevalence estimation: Method to improve intervention coverage estimations. Proceedings of the National Academy of Sciences. 2018;115(51):13063-8.

3.	Amoakoh-Coleman M, Kayode GA, Brown-Davies C, Agyepong IA, Grobbee DE, Klipstein-Grobusch K, et al. Completeness and accuracy of data transfer of routine maternal health services data in the greater Accra region. BMC Res Notes. 2015;8:114. Epub 2015/04/19. doi: 10.1186/s13104-015-1058-3. PubMed PMID: 25889945; PubMed Central PMCID: PMCPMC4392754.

4.	Xiao Y, Bochner AF, Makunike B, Holec M, Xaba S, Tshimanga M, et al. Challenges in data quality: the influence of data quality assessments on data availability and completeness in a voluntary medical male circumcision programme in Zimbabwe. BMJ Open. 2017;7(1):e013562. Epub 2017/01/31. doi: 10.1136/bmjopen-2016-013562. PubMed PMID: 28132009; PubMed Central PMCID: PMCPMC5278271.

5.	Muthee V, Bochner AF, Osterman A, Liku N, Akhwale W, Kwach J, et al. The impact of routine data quality assessments on electronic medical record data quality in Kenya. PLoS One. 2018;13(4):e0195362. Epub 2018/04/19. doi: 10.1371/journal.pone.0195362. PubMed PMID: 29668691; PubMed Central PMCID: PMCPMC5905951.

6.	Evaluation M. Routine Data Quality Assessment Chapel Hill: Measure Evaluation; 2019 [cited 2021 3 December 2021]. Available from: file:///Users/joevaladez/iCloud%20Drive%20(Archive)/Documents/publications/Annealing/Alex's%20paper/PLoS%20Global%20Health/Revision/tl-19-76.pdf.
